# Computing expectation values for RNA motifs using discrete convolutions

**DOI:** 10.1186/1471-2105-6-118

**Published:** 2005-05-13

**Authors:** André Lambert, Matthieu Legendre, Jean-Fred Fontaine, Daniel Gautheret

**Affiliations:** 1 CNRS UMR 6207, Université de la Méditerranée, Luminy Case 907, 13288 Marseille cedex 9, France; 2 INSERM ERM 206, Université de la Méditerranée, Luminy Case 928, 13288 Marseille Cedex 9, France; 3 INSERM EMI U 00.18, CHU d'Angers, 49033 Angers, France

## Abstract

**Background:**

Computational biologists use Expectation values (E-values) to estimate the number of solutions that can be expected by chance during a database scan. Here we focus on computing Expectation values for RNA motifs defined by single-strand and helix lod-score profiles with variable helix spans. Such E-values cannot be computed assuming a normal score distribution and their estimation previously required lengthy simulations.

**Results:**

We introduce discrete convolutions as an accurate and fast mean to estimate score distributions of lod-score profiles. This method provides excellent score estimations for all single-strand or helical elements tested and also applies to the combination of elements into larger, complex, motifs. Further, the estimated distributions remain accurate even when pseudocounts are introduced into the lod-score profiles. Estimated score distributions are then easily converted into E-values.

**Conclusion:**

A good agreement was observed between computed E-values and simulations for a number of complete RNA motifs. This method is now implemented into the ERPIN software, but it can be applied as well to any search procedure based on ungapped profiles with statistically independent columns.

## Background

The introduction of the Expectation value (E-value) in the Blast program in 1990 [1] was a major milestone in the development of sequence search algorithms. For any sequence match with a score *S* obtained in a given database, the E-value is the number of hits of same or higher score that can be expected by chance. E-values tell biologists how likely they are to encounter a specific sequence match in a database search, which is a more useful view of biological significance than a mere similarity score. Except in some special cases, low E-values are commonly accepted as an evidence of sequence homology.

The recent years have seen a growing interest for RNA motif searches, driven by the discovery of important new classes of regulatory RNA genes and motifs in all organisms. Non-coding RNAs are characterized in a large part by long range base pair interactions, whereas linear sequence constraints are not as important as in protein coding genes. As a result, standard sequence alignment programs such as Blast are not suited to RNA motif search. Computational biologists have addressed this problem in several ways, notably through descriptor-based systems in which the topology of base paired regions is user-specified [3,5,4], Stochastic Context Free Grammars (SCFG) which use a complete statistical model of RNA elements [6], and Secondary Structure Profiles, which use position weight matrices describing stems and single strands in the RNA motif, as found in the ERPIN program [8]. Although the last two methods compute alignment scores, the complexity of the underlying models and search algorithms has hampered the estimation of an E-value to date.

When the behavior of the score distribution is known, such as in sequence alignment scores, E-values can be computed either directly, or by empirically fitting a histogram of scores from a sample of random sequences to the assumed distribution function [1,2]. Unfortunately, we will show here that a search algorithm such as the one used in ERPIN does not produce predictable score distributions. A possible workaround for this practical limitation is to run simulations on randomized sequences and use the observed hit count at score *S *as the E-value for this score. However, since interesting high scoring solutions can be extremely rare (commonly less than one random occurrence per genome) simulations often require days of calculations. Can we then estimate score distributions without having to run such lengthy simulations?

In this article, we show that ERPIN score distributions can be estimated a priori through a discrete convolution analysis of score profiles, based on a random model of nucleotide frequencies. This led us to develop a computational procedure that estimates the score distribution of ERPIN profiles in a very short time, before the actual search begins, so that each solution can be automatically assigned an E-value.

## ERPIN profiles

**Figure 1:**
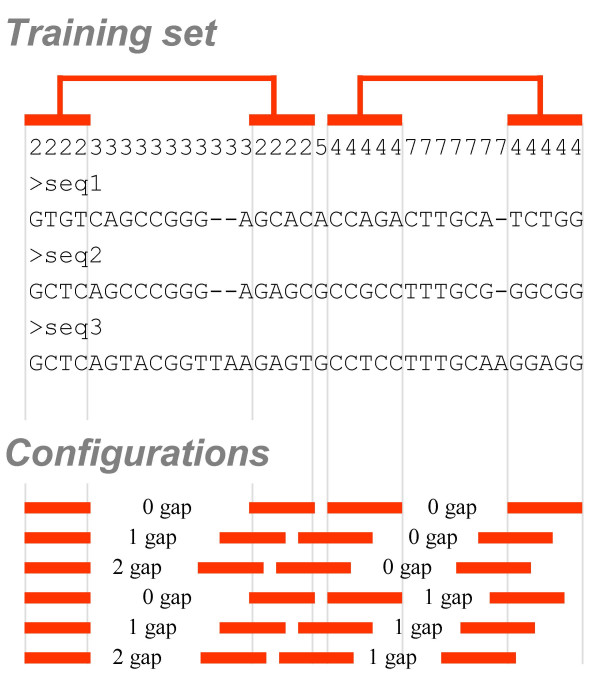
An example of ERPIN training set containing two double helices (noted 2 and 4), and three single strands (noted 3, 5 and 7). Due to gaps in the alignment, helix 2 spans 9 to 11 nt, and helix 4 spans 6 to 7 nt. Combinations of these allowed ranges give 6 possible configurations for the whole RNA motif.

ERPIN is an RNA motif search software using as input a training set of aligned RNA sequences, and a target database in which the motif is to be identified. The training set contains both RNA sequence and their common secondary structure, specified as shown in Figure 1 in the form of single strands and helices. Importantly, gap characters (insertions or deletions) are allowed in single strands but not in helices. Helices are composed of two distinct strands of equal length. A region is defined as a continuous stretch of complete single strands or helical elements. When only one strand of a helix is included in a region, this strand is considered as a single strand. A mask is a subset of a region constituted of single strands and/or complete helices.

ERPIN converts each helix and single strand in the alignment into a lod-score profile. This involves two steps. First, columns in the alignment are converted into frequency profiles, recording the frequencies of bases or base-pairs in column *c *as:



Here *n*_*ic *_is the number of bases or base-pairs of type *i *in column *c*, and *N *is the total base or base-pair count in a column. There is one frequency profile for each single strand or helix in the alignment. In the case of a single strand, *i *∈ {*A*, *T*, *G*, *C*}, whereas for a helix, *i *∈ {*AA*, *AT*, *AG*, *AC*, ..., *CC*} and each column in a helical profile actually refers to two positions in the initial alignment. Single strand profiles thus have 4 rows while helix profiles have 16 rows. The special case of gap-containing single strands, where a fifth character is added to the profile is discussed later on.

Frequency profiles are then converted into lod-score profiles, where values for column *c *are defined as:



where *b*_*i *_is the background frequency of base or base-pair *i *in the target database. Base-pair frequencies are considered as the product of individual base frequencies. The score of a helix or single-strand element is obtained by presenting a target sequence to this element's profile and summing the scores obtained for every profile column. For ungapped elements, the calculation is straightforward. For gap-containing single strands, a dynamic programming matrix of the profile and target sequence is constructed, which provides the best possible alignment score [8]. In a first stage, let us ignore this alignment procedure and focus on ungapped elements.

### Exclusions and pseudocounts

We define as an exclusion a profile element for which no base or base pair is observed in the training set, and thus *P*_*ic *_= 0. Exclusions may be due either to an unsufficient size of the training set, or to a true avoidance of this particular base or base-pair at this position in the RNA molecule. In any case, the log-odd ratio formula would produce a value of -∞ for such cases, thus requiring a special treatment. Exclusions are dealt with either by using arbitrary low values (e.g. -30) or by introducing pseudocounts in the frequency matrix that simulate what could have been observed in a larger training set.

Pseudocounts are based on some prior knowledge of "typical" substitution frequencies in RNA molecules, as observed in a model RNA sequence alignment. The pseudocount calculation procedure used in ERPIN is the same spirit as that of Henikoff and Henikoff [9], but we use a different definition of pseudocounts, as explained below. Let us first reformulate the values in any column *c *of a frequency profile:



Where *n*_*ij *_is the number of {*i*, *j*} couples in column *c*, *P*(*i*, *j*) is the joint probability of finding *i *and *j *in this column, and . This develops into:



Where *P*(*i*|*j*) is the conditional probability of observing *i*, knowing that *j *is observed in the column. This conditional probability amounts to the observed frequency of *i *→ *j *substitutions. To introduce pseudocounts in ERPIN frequency profiles, *P*(*i*|*j*) is replaced with the average substitution frequencies observed in a model RNA sequence alignment, expressed in the form of a substitution matrix *M*. Pseudocount-based frequencies can be expressed as:



where *M *is a square matrix whose columns *j *are normalized, ∑_*i *_*M*_*ij *_= 1, so that . See Methods section for construction of *M*. The relative ratio of pseudocounts to true counts in the final frequency matrix is then controlled by a user-defined weight parameter *α *∈ [0, 1], such that:



Since *M*_*ij *_are generally ≠ 0 in the substitution matrices (either for single strand or helices), most exclusions in the frequency profile are replaced by nonzero values as soon as *α *≠ 0. Not only the resulting lod-score profiles are basically devoid of arbitrary low values, but they better reflect "natural" base and base-pair substitution frequencies observed in real RNA alignments. This is especially interesting in helical regions, since the substitution matrix for helices represent natural exchanges between frequent base-pairs such as Watson-Crick, G:U or even G:A, while incurring strong penalties for exchanges involving rare base pairs. This maintains a large fraction of strongly negative values in helix profiles, which is desirable for the sake of search specificity.

## Shapes of score distributions: finite and non-finite scores

We define as a "finite" score the score obtained for a sequence that does not contain any match to a profile exclusion. When pseudocounts are used, almost all scores are finite, but when pseudocounts are not in use (*α *= 0), many scores, especially in helix profiles, are "non-finite", although in practice they are replaced by arbitrary low values.

Let *S *denote a finite score obtained at a given site in a random sequence. For any *x *> -∞, the conditional probability formula reads:

*P*(*S *> *x*) = *P*(*S *> -∞).*P*(*S *> *x*|*S *> -∞)     (7)

In this decomposition, it is noteworthy that:

• The first factor is independent of *x *as soon as *x *is finite,

• The second factor can be computed based on profile elements that contain no exclusion.

Let *S*_*c *_(*c *= 1, 2, .., *w*) denote a score for column *c *of a profile, and *S *= *S*_1 _+ *S*_2 _+ ... + *S*_*w *_the score obtained by presenting a given sequence to this profile. If *w *is large enough (*w *≳ 10) and the distributions of random variables *S*_*c *_are (i) independent and (ii) identically distributed, the sum *S *follows a normal distribution (central limit theorem [11]).

**Figure 2:**
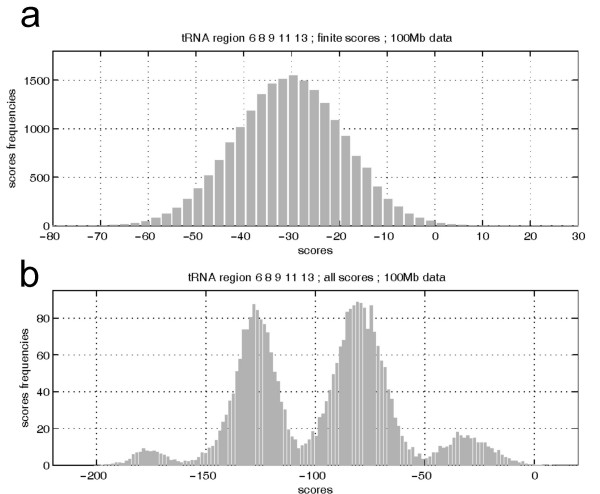
Distributions of finite and total scores obtained from the motif encompassing the anticodon and *T*Ψ*C *loop of tRNA, at each position of a 100 Mb random sequence database. This region covers three single strand profiles and two helix profiles and spans a gap-containing single- strand profile that is not included in score calculation. ERPIN results were processed by the *epnstat *utility program. A: finite scores. B: total scores (both finite and non-finite).

Due to exclusions arising with different frequencies in different columns, condition (ii) is generally not fulfilled, but, using the decomposition given by formula (7) rewritten for column *c*, we can write:

*P*(*S*_*c *_> *x*) = *P*_*fs*,*c*_.*P*_*f*,*c*_(*x*)     (8)

where *P*_*fs *_is the probability that a score is finite, and *P*_*f*_(*x*) is the probability that a finite score is higher than *x*. This gives, for a complete profile:



In many cases, the probability distributions of finite scores *P*_*f*,*c *_are similar enough so that their sum *S *is normally distributed. If this behavior was always observed, the score distribution could be fully determined by computing the mean value *μ *and the standard deviation *σ *that characterize the normal law, which would enable a direct calculation of the E-value [2]:



Figure 2 shows the score distributions obtained using the tRNA region spanning the anticodon and *T*Ψ*C *loop (two helices + three single-strands) at each position of a 100 Mb random sequence database, with pseudocounts switched off (*α *= 0). As expected, finite scores (Fig 2a) follow a normal distribution, while total scores (Fig 2b) are unevenly distributed.

So called "non-finite" scores may be biologically relevant, since many valid substitutions are potentially absent from the training set. However, non-finite scores are detected only when high enough to fall into the extreme end of the distribution. This part of the distribution is composed mainly of finite scores and should thus behave like that of finite scores. Unfortunately finite scores may also deviate from a Gaussian distribution, for instance when score distributions in successive columns are too different from each other.

**Figure 3:**
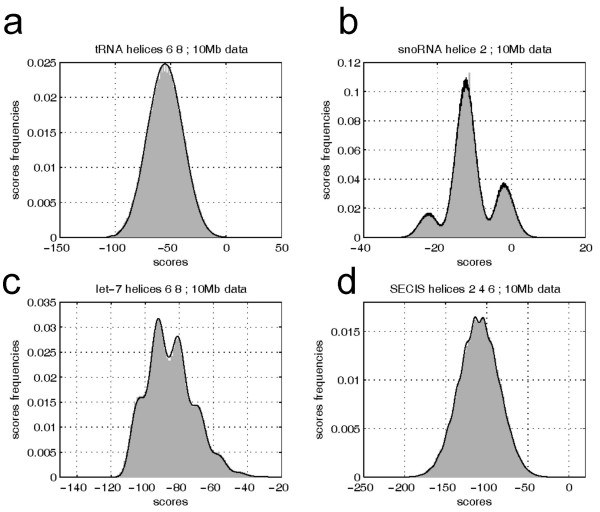
Comparision of finite score distributions obtained from discrete convolution of helix profiles (solid lines) and simulation (shaded bars). The various helices in the region under study were combined into a larger 16 × *W *profile, where *W *is the total number of base-pairs in the region. Lod-scores were computed based on a uniform nucleotide composition, by the *convhstat *utility program.

**Figure 4:**
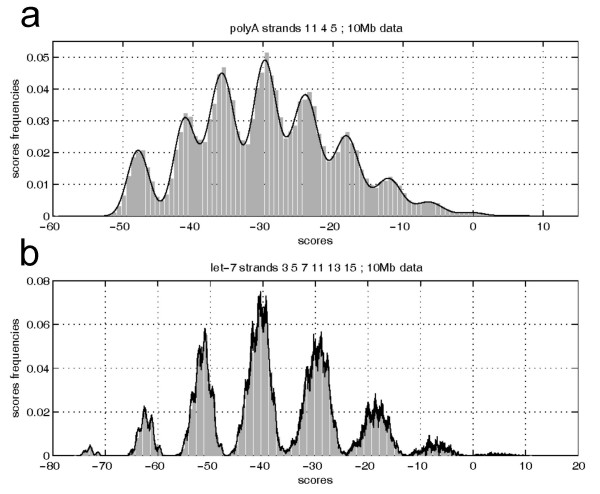
Comparision of finite score distributions obtained from discrete convolution of single-strand profiles (curve) and simulation (shaded bars). The various strands in the region under study were combined into a larger 4 × *W *profile, where *W *is the total number of nucleotides in the region. Lod-scores were computed based on a uniform nucleotide composition, by the *convsstat *utility program.

Pseudocounts are used to inject missing substitutions into frequency profiles, resulting in most "non-finite" scores becoming "finite". But what is the behavior of finite scores when pseudocounts are in use? Fig 3a–d and 4a–b show finite score distributions for a variety of ungapped RNA motifs (shaded bars), obtained using a typical level of pseudocounts (*α *= 2.10^-4^) in frequency profiles. Although some training sets (tRNA, SECIS) have nearly gaussian distributions, others (let-7 miRNA, snoRNA, polyA sites) are more erratic. This is due to the larger number of exclusions in the later sets – only partially compensated for by pseudocounts – and/or their non-uniform distribution over profile columns. If we aim to address true biological problems with such imperfect or sparse training sets, we necessarily have to deal with this type of score distribution that cannot be approached with classical methods. Nonetheless, we will still be using the decomposition formula (7), as it provides an important reduction of the range of values of the random variables involved.

## Score distributions of helices and single-strands

### Ungapped helices and single strands

How can we estimate score distributions such as those in Fig 3, 4 ? Let us admit that profile columns are independent. After matching the profile to a purely random sequence, the resulting scores for each column would thus behave as independent random variables, say *X*_1 _and *X*_2 _for columns 1 and 2. Therefore, the final score for two columns would be:

*S *= *X*_1 _+ *X*_2_

Then, the probability of obtaining a score *S *= *x *is:



The last formula defines the *discrete convolution product *[11] of two distributions. The overall score distribution can be obtained by doing the calculation for every possible values of *u *and *v*.

Using the separation formula (7) between "exclusions" and "finite scores", equation (10) can be written:



These operations can easily be extended to *N *columns by iterating the products on successive columns in the same single-strand or helix profile. At each successive iteration, scores are discretized on a predefined grid so that the number of possible scores increases linearly with the number of columns (see Methods section for algorithm).

We performed such an analysis on a variety of helix and single strand profiles, with grid intervals set at Δ*x *= .05. In Figures 3 and 4, score distributions estimated from discrete convolution (solid lines) are compared to scores obtained through simulation on a random database of variable size (shaded bars). There is a very good agreement between the discrete convolution and simulation.

### Gap-containing single strands

The score of a gap-containing single strand in the ERPIN program is computed from the dynamic programming alignment matrix. Therefore, it is the maximum of several values, and could be expected to comply with an extreme value distribution. However, gapped single strands in ERPIN are very diverse entities that may include oddities such as single-nucleotide strands, or strands mostly filled with gaps. This results in very uneven distributions that we were not able to model satisfyingly. Therefore, the score distribution of a gapped single strand in ERPIN is currently estimated based on a short simulation performed on a random sequence (see Methods section for details).

## Score distributions of complete regions with gaps

### Score of a configuration

When presenting a sequence to a whole region, the presence of gaps in single-strands results in multiple allowed positions for flanking helical elements. A configuration is a specific arrangement of helix elements determined by the number of intervening gaps (Fig 1). There is one score for each allowed configuration, which is the sum of scores for all helices and single strands in this configuration. We therefore need to compose the different score distributions to obtain the distribution of the total score for one configuration. This is again done using a discrete convolution of these distributions, with the same procedure and grid parameter as above. This provides the score distribution of a single configuration. Note that, although a configuration may contain gapped single strands of which score distribution was not produced by a discrete convolution, such distributions can now be treated by this second convolution round applied to whole profiles.

### Score of a complete region

The number of gaps in a single strand is bounded by the maximum number of gaps observed for this strand in the training set: *mxgaps*. For a simple hairpin-loop motif with *mxgaps *possible gaps in the loop, there are (*mxgaps *+ 1) possible configurations. For a whole region containing *N *strands with gaps (*i *= 1, 2, ..., *N*), the number of configurations is:



Erpin evaluates all possible configurations without any construction rule or strategy. A combinatorial explosion is avoided by implementing multi-stage searches, where search at each stage is limited to a defined mask, or subset of the region under study. The score of a region or mask at in given site is the maximum score obtained for all possible configurations.

Since a motif is identified only after all possible configurations are evaluated at a given site, our estimation of motif scores requires taking into account this additional complexity. Let *K *denote the number of configurations for a given motif, and *S*_*i*_, the score obtained for the *i*th configuration. As the ERPIN program does not permit the of addition gaps relative to those present in the training set, *K *is necessarily bounded but its value can be relatively large. We are now interested in the maximal score obtained for all configurations at each site. This is the extreme value distribution, or the distribution of a random variable *M *defined as:



If *P*_*fs *_= *P*(*S*_*i *_> -∞) and *p*_*i*_(*x*) = *P*(*S*_*i *_> *x*|*S*_*i *_> -∞), and the random variables *S*_*i *_are statistically independent (*s.i*) and identically distributed (*i.d*), then:





The most "interesting" scores are expected to be of the same order of magnitude as those obtained by training set sequences. For any realistic training set, these scores should be very high compared to scores obtained on random sequences and, therefore, their probability should be very low. In this case *P*(*M *> *x*), given by formula (17), behaves at the first order approximation as *K.P*_*fs*_.*p*(*x*).

For a database of size Ω, considering that individual sequences in the database are large enough compared to the search motif so that border effects can be ignored, the final E-value, is :

*E*(*x*) = *P*(*M *> *x*).Ω     (18)

**Figure 5:**
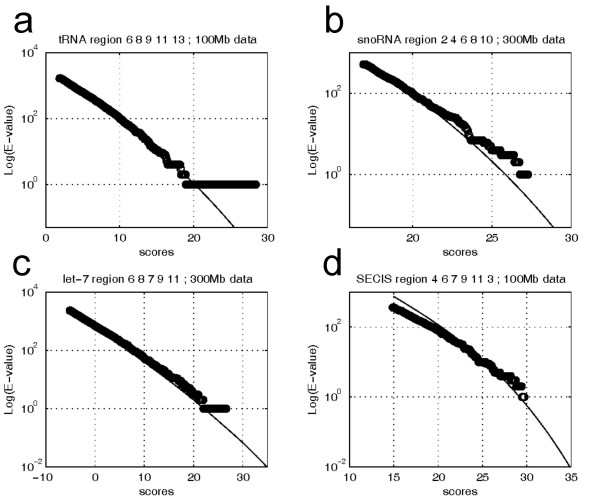
Comparision of computed E-values (solid lines) and number of solutions obtained from simulation on a random database of uniform nucleotide composition (circles), for different RNA motifs. Numbers following "region" refer to secondary structure elements in the corresponding training set available from . E-values were computed using the *mstat *utility program. (a) tRNA region covering the anticodon and *T*Ψ*C *stem-loops; (b) C/D box snoRNA region covering the major stem and C+D boxes; (c) Let-7 miRNA region covering the complete precursor hairpin; (d) SECIS element covering the large 14 bp stem and apical stem+loops.

Figure 5 compares these computed E-values (solid lines) to simulations performed on a random database (circles), for complex RNA regions encompassing multiple helices and singles strands (gapped or ungapped). Overall there is a very good agreement between E-value and simulation, consistent with our hypothesis that configuration scores are independent and equally distributed. Importantly, E-values remain accurate for RNA regions containing large gapped single strands, such as snoRNA (Fig 5b), Let-7 miRNA (Fig 5c) and SECIS (Fig 5d), and over a wide range of scores. This last point is also important, since "borderline" solutions with an E-value around 0.1 or 1 are potentially more interesting biologically as low E-value solutions. Moreover, computing times for overall E-value calculations in all our tests motifs remained insignificant relative to database scan times.

In the case where pseudo-counts are switched-off, profiles contain multiple "non-finite scores" which are excluded from the convolution process. This may imply a lack of accuracy in the left-hand side of the estimated score distribution, where scores have low values, but should have little effect in the region of biologically interesting scores. Therefore we do not expect E-values to deteriorate significantly in practise when pseudocounts are switched off.

## Conclusion

We have presented a method to estimate the score distributions of RNA helices or single strand profiles and of their combinations into larger motifs. This method is based on discrete convolutions. The computing time of the discrete convolution algorithm increases quadratically with profile size and remains in any case negligible relative to database scan durations. This procedure is implemented in the last release of the ERPIN software (V. 4.2) and provides accurate estimates of E-values for practical applications. Interestingly, the discrete convolution approach can be applied as well to others sequence scoring models -nucleic acids or proteins – based on ungapped profiles with independent columns.

## Methods

### ERPIN program and utilities

The ERPIN program (sources and executables) is available at . Simulated score distributions of independent helix and single-strand profiles (Fig 2, 3, 4) were obtained using the -*hist *(histogram) option of ERPIN and utility programs *epnstat*, *convhstat*, *convsstat *and *mstat *provided in the distribution. For Fig 5, full motif searches were performed using ERPIN Version 4.2.5 with pseudocount weight *α *= 2.10^-4^. Graphical outputs for figures 2, 3, 4, 5 were produced using the Matlab [13] package.

### Training sets

Training sets for profile statistics and ERPIN runs in Fig 2, 3, 4, 5 are available on the ERPIN web site and were obtained as follows:

• tRNA: 903 type I tRNA sequences (all species) from the 1997 version of M. Sprinzl's nuclear tRNA alignment [17].

• SECIS (Selenocystein Insertion Sequence): 117 metazoan SECIS sequences, from our own compilation [7].

• snoRNA (Small nucleolar RNA): 217 archaean C/D box snoRNA sequences, compiled and aligned by Fabrice Leclerc at CNRS Nancy (Personal communication).

• Let-7 miRNA: 27 animal miRNA precursor sequences from our previous compilation [15].

• PolyA sites: 2327 human polyadenylation sequences from our previous compilation [16].

### RNA substitution matrices for pseudocounts

Pseudocount calculation requires substitution matrices obtained from a model RNA sequence alignment or "training set", annoted with secondary structure information (helix or single strand). Klein and Eddy have developed RNA substitution matrices previously [18], but we use a different type here. Training set columns are converted into profiles containing raw nucleotide or base-pair counts. Let *Q *denote a nucleotide or base-pair count profile of width *w*, produced by a concatenation of all single strand or helix profiles from the pseudo-count training set. *Q *is either a 4-line matrix for single strands (*h *= 4) or a 16-line matrix for helices (*h *= 16). The substitution matrix *M *introduced in Section "Exclusions and Pseudocounts" is then a square matrix of size *h *× *h *defined as:







The square matrix *N *is actually a "correlation matrix" of the profile lines since element *N*_*ij *_is the scalar product correlation of lines *i *and *j*. Coefficients *λ*_*j *_make this matrix normalized, so that:



Probability conservation is verified for *P *and therefore it is also verified for *P'*:



Finally, it is obvious from formula (6) that *P" *also verifies verifies probability conservation, hence:



Substitution matrices can be generated from any RNA sequence alignment using the utility program *mksum *of the ERPIN distribution. Default matrices provided with distribution (*SUM.dat *file) were obtained using a 16S/18S rRNA training set from R.Gutell ([10]) containing 6310 sequences from all three phylogenetic domains. We used the secondary structure of E-coli 16S rRNA as the consensus structure, resulting in 481 columns of helix profile and 7512 columns of single strand profile.

Option -*pcw *is used to set pseudo-count weight *α *in the ERPIN program. Default internal value is 2.10^-4^, but this has been rescaled for users by a factor of 2.10^-3 ^giving a default user value of 0.1 and a practical maximal value that should not exceed 1. Effects of pseudocounts and of the *α *parameter on profiles can be visualized using the utility program *pview*.

### Score distribution of gap-containing single-strands

The score distribution of gap-containing single strands is evaluated by repeatedly computing profile scores with a random sequence of same length *L *as the profile and same composition as the target sequence database. The calculation is repeated *C.L*^2 ^times, with *C *a constant, and *L *<*L*_*max *_in order to limit CPU time for unusually large strands. Default values are *C *= 300 and *L*_*max *_= 12.

### Histograms and discrete convolution product

Although discrete convolutions can be computed using iterated Fast Fourier Transforms, this approach is subject to numerical approximations in practice. A direct calculation is more accurate and proved fast enough in all cases tested. Time complexity of the discrete convolution algorithm is *O*(*N*^2^) where *N *is the total number of profile columns. This value remains tractable even for the largest RNA motifs. The convolution algorithm was adapted from those found in the Octave [12] and Matlab [13] packages. The linear sampling interval was set at Δ*x *= .05. CPU time for the complete E-value calculation (including profile construction, convolution of independent profiles and convolution of configurations) for motifs in Fig 5 ranged from 10^-4^s to 0.8s on a 2.6 GHz Intel Pentium workstation with 1 Gb of RAM.

### Extreme value distribution

Formula (17) used for calculating the extreme value distribution is of the type (1 - (1 - *x*)^*N*^). If *x.N *> 1 the result is obtained with the C library function *x **x*^*y *^which lacks precision when *x *≪ 1. Otherwise we compute (17) using the binomial formula for (1 - *x*)^*N*^.

## Authors' contributions

AL conceived the discrete convolution approach, programmed the software and participated in drafting the manuscript, ML and JFF performed program runs and statistical analyzes of outputs, DG participated in the design and coordination of the study and wrote most of the manuscript. All authors read and approved the final manuscript.
